# Brief Educational Workshops in Secondary Schools Trial (BESST trial), a school-based cluster randomised controlled trial of the DISCOVER workshop for 16–18-year-olds: recruitment and baseline characteristics

**DOI:** 10.1186/s13063-024-08116-7

**Published:** 2024-05-04

**Authors:** Kirsty James, Stephen Lisk, Chloe Payne-Cook, Zamena Farishta, Maria Farrelly, Ayesha Sheikh, Monika Slusarczyk, Sarah Byford, Crispin Day, Jessica Deighton, Claire Evans, Peter Fonagy, David Saunders, Irene Sclare, James Shearer, Paul Stallard, Timothy Weaver, Jynna Yarrum, Ben Carter, June S. L. Brown

**Affiliations:** 1 King’s College London, Institute of Psychiatry, Psychology and Neuroscience, King’s College London, De Crespigny Park, London, SE5 8AF UK; 2 Department of Biostatistics and Health Informatics, & King’s Clinical Trials Unit (KCTU), Institute of Psychiatry Psychology and Neuroscience, King’s College London, Denmark Hill, London, SE5 8AF UK; 3 University of Bath, Bath, UK; 4 University of Northampton, Northampton, UK; 5 Anna Freud Centre for Children and Families, London, UK; 6 Southwark CAMHS Clinical Academic Group, South London and Maudsley NHS Foundation Trust, London, UK; 7 Department of Clinical Educational and Health Psychology, University College London, London, UK; 8 Middlesex University, London, UK

**Keywords:** Mental health, Adolescents, School based, Open access, Baseline, Recruitment

## Abstract

**Background:**

The Brief Educational Workshops in Secondary Schools Trial (BESST) is an England-wide school-based cluster randomised controlled trial assessing the clinical and cost-effectiveness of an open-access psychological workshop programme (DISCOVER) for 16–18-year-olds. This baseline paper describes the self-referral and other recruitment processes used in this study and the baseline characteristics of the enrolled schools and participants.

**Method:**

We enrolled 900 participants from 57 Secondary schools across England from 4th October 2021 to 10th November 2022. Schools were randomised to receive either the DISCOVER day-long Stress workshop or treatment as usual which included signposting information. Participants will be followed up for 6 months with outcome data collection at baseline, 3-month, and 6-month post randomisation.

**Results:**

Schools were recruited from a geographically and ethnically diverse sample across England. To reduce stigma, students were invited to self-refer into the study if they wanted help for stress. Their mean age was 17.2 (SD = 0.6), 641 (71%) were female and 411 (45.6%) were from ethnic minority groups. The general wellbeing of our sample measured using the Mood and Feelings Questionnaire (MFQ) found 314 (35%) of students exhibited symptoms of depression at baseline. Eighty percent of students reported low wellbeing on the Warwick Edinburgh Mental Wellbeing Scale (WEMWBS) suggesting that although the overall sample mean is below the cut-off for depression, the self-referral approach used in this study supports distressed students in coming forward.

**Conclusion:**

The BESST study will continue to follow up participants to collect outcome data and results will be analysed once all the data have been collected.

**Trial registration:**

ISRCTN registry ISRCTN90912799. Registered on 28 May 2020.

## Introduction

Most adult affective disorders emerge before adulthood [1, 2], causing distress to young adults. The consequences of depression (including sub-threshold levels) within teenagers are poorer social, educational, and occupational outcomes [3–5] and long-term mental illness. However, less than a quarter are known to specialist child and adolescent mental health services (CAMHS) in the UK [6]. Barriers to accessing specialist mental health support include inconvenience, transportation to services, long waiting lists and a high threshold of referral from primary care [7, 8]. Even when young people have presented to CAMHS there is limited access to effective interventions such as cognitive-behavioural therapy (CBT) and other evidence-based psychological therapies [9]. To address these problems, the UK government set up a new system of mental health support teams (MHSTs) [10] whereby a new workforce, based in schools, are trained to deliver low intensity CBT approaches to address mild to moderate symptoms of anxiety, depression, and behaviour difficulties.

The DISCOVER day-long workshop programme [11] was developed for school settings to reduce stress. The programme was adapted from the adult version of the workshop [12] and consists of an initial goal-setting session, a 1-day CBT workshop and up to 3 follow-up phone calls to focus on individual goal setting.

Given the low effectiveness of universal interventions [13] and the stigma experienced by targeted participants [14], we are using a ground-breaking participant-led self-referral system used by Brown [15] where the individuals decide if they want to be involved by referring themselves. This has several major advantages. It increases accessibility to anyone who identifies themselves as experiencing problems, emphasises autonomy, which is valued by adolescents, and allows economical use of resources. In prior research, this approach has led to high engagement by students who have not previously sought help and led to high follow-up rates of over 90% [16].

Secondly, the novel self-referral system has been shown to attract a higher proportion of people from ethnic groups in the community [16, 17] to engage in interventions. This is particularly important given that people from ethnic minorities are underserved in mental health treatment and research despite often higher need [18]. In addition, figures on people from ethnic groups are often not recorded, and even if they are, are not reported [19]. Minority ethnic groups often find it difficult to access services [20], meaning that participants in research studies end up being largely white British [18].

In previous work, we conducted a cluster randomised controlled feasibility trial of DISCOVER versus usual care (1:1). We enrolled 155 students, aged 16- to 18-year-olds, from 10 schools in London. The majority were female (81%) and 57% were from ethnic minorities. Of those that enrolled, 72% attended the full day workshop with a significant reduction in depression (*d* = 0.27) and anxiety (*d* = 0.25) reported at 3 months after baseline.

The protocol for the BESST study has been published [21] so further information about rationale for the study can be found there. The purpose of this cohort profile paper is to provide detail on the recruitment process for this large multi-site cluster randomised trial in a secondary school setting. Baseline data from the recruited schools and participants will also be provided to characterise our sample.

## Methods

### Study design

BESST is a cluster randomised controlled trial (cRCT) (ISRCTN registry ISRCTN90912799, registered with ISRCTN 28 May 2020). Schools from 15 geographical areas across four regions of England were invited to participate in the study. The primary and secondary outcome measures were assessed at baseline, 3-month, and 6-month post randomisation. The researchers collecting the post-baseline measures, chief investigator and senior analyst were blinded to the trial arm throughout the trial.

### School recruitment

School recruitment took place in four regions in England, the Northwest, West Midlands, London, and the Southwest. Recruitment was over two school years (2021/2022 and 2022/2023), aligning with the start of the school academic years, with intake after the summer holidays in order to follow up participants at 6 months prior to the end of the year exam period in May. School recruitment began in September 2021 and closed in December 2021 for year 1 and took place between September 2022 and December 2022 for year 2.

Schools were approached in regions served by the local MHSTs and were identified via two methods: an introduction to a relevant school staff member was made via the MHST, after which members of the research team would meet to discuss the trial with the school staff. Alternatively, and less frequently, the research team would identify and contact the sixth-form staff in appropriate schools directly and follow the same process if interest was indicated.

One hundred and eleven state-funded schools containing a sixth form (students aged 16–18 studying A levels) were first identified through either MHST referral or searching the local records. These schools were first screened for eligibility prior to contact being made; they were then formally screened once they had expressed interest in participating in the trial. In order to be eligible for the study schools must be (i) secondary schools with sixth form or dedicated sixth form college, (ii) state-funded and have (iii) sufficient resources available to host the trial. Exclusion criteria were (i) further education college, (ii) privately funded school/college and (iii) sixth form student population < 70.

One hundred and four eligible sixth forms were approached to take part in the study. They were asked to express written interest in the study, after which a final screening for eligibility was conducted, and an information session was organised to explain the study to the relevant staff members at each school and 57 consented into the trial. The necessary school level summary characteristics were sent to the statistician to carry out the randomisation of the block of schools for each area. Following this, dates were organised with the school for the site trial Research Assistant (RA) to attend a sixth-form assembly and present the study to the students. Any interested students were invited to attend a lunchtime information session where they were able to find out more information about participation and receive a written information sheet and consent form. Students were asked to review the information and inform a designated member of school staff by a set date if they would like to participate.

### Participant recruitment

From the 57 consented schools, 1407 students attended the project information meetings, of which 991 went on to consent to the study and were screened for eligibility. Once consented, the list of students was sent to an independent statistician to randomly generate a list to determine which 19 students from each school would be invited to take part in the study and be screened for eligibility. This was limited to 19 students per school for practical reasons in implementing the DISCOVER workshop. Participant inclusion criteria were (i) aged between 16 and 18 years, (ii) attending school or college, (iii) sufficient English to provide valid informed consent and complete assessments in the BESST study, (iv) seeking psychological help for stress, (v) able to attend the DISCOVER workshop on school premises and (vi) able to provide informed written consent to participate. Exclusion criteria were (i) identified as actively suicidal through risk assessment, (ii) current involvement in psychological therapy for anxiety or depression with CAMHS, or (iii) severe learning difficulties. The 926 eligible participants were then invited to take part in the study and complete baseline assessments. Once all baseline assessments had been completed, the allocation for that school was released and the school was informed.

**Fig. 1:**
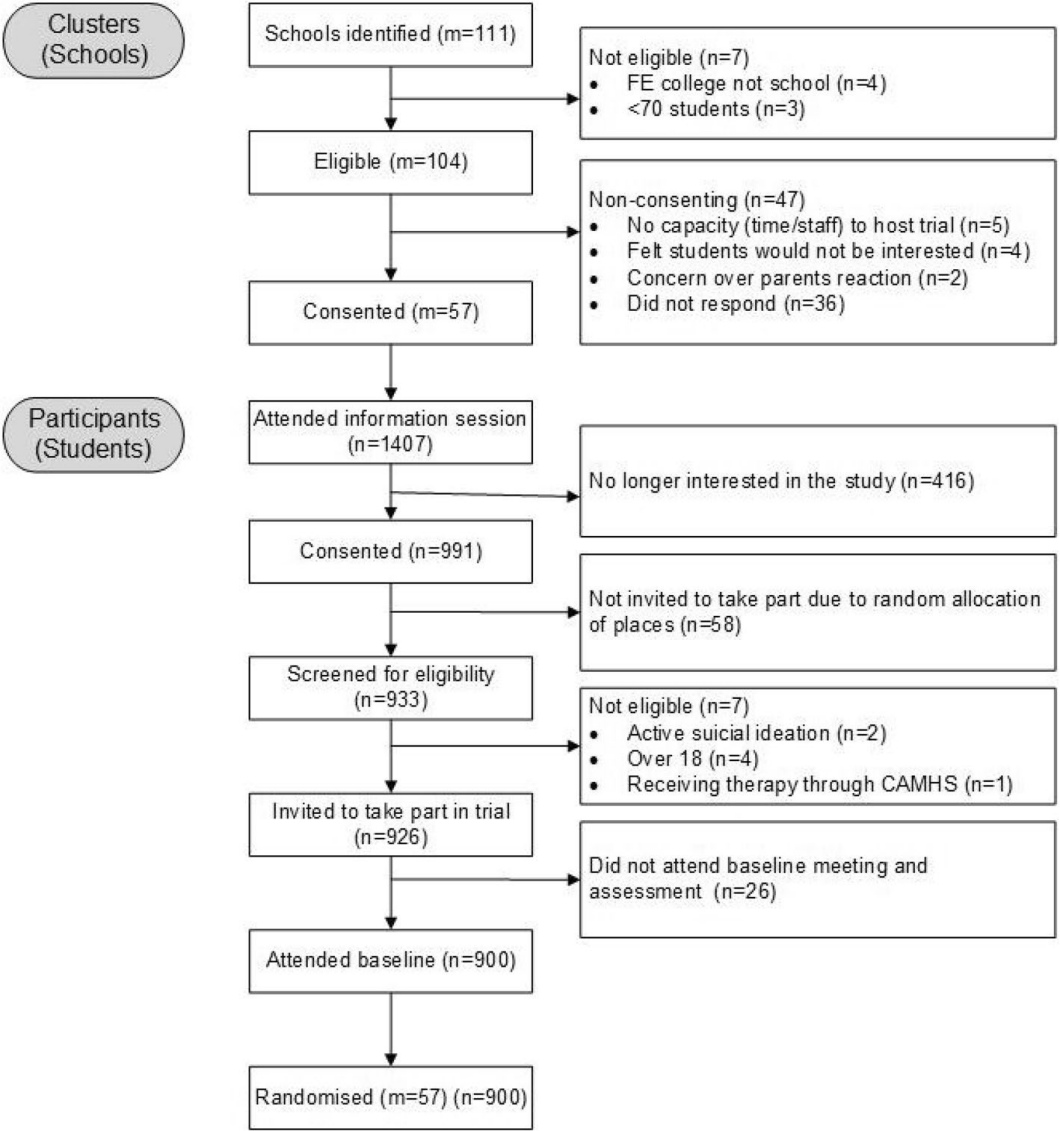
CONSORT diagram

Full flow of schools and participants recruitment into the study can be seen in Fig. 1.

### Randomisation

Randomisation was done at school level in a 1:1 ratio. The sequence was generated by a statistician not within the research team using a randomisation algorithm for cluster randomised trials developed by Carter [22] and was stratified by site with balancing covariates of school size and index of multiple deprivation (IMD) [23] 

### Data collection

Primary, secondary, and economic measures shown in Table 1 were collected at the participant level at baseline, 3-month, and 6-month follow-up. Full information about the outcome measures can be found in the protocol [21]. The primary outcome measure for this study is the Mood and Feelings Questionnaire (MFQ). The MFQ is a 33 item self-report depression measure with scores ranging from 0 to 66 with a higher score indicating a higher level of depression. Baseline and follow-up assessments took place in schools and were facilitated by local/site RAs. The RA arranged a 1–2–1 appointment with each participant, via relevant school staff. The appointment took place in a private room at the participants’ school, where the RA explained each measure to the participant and then allowed the participant to complete the measures whilst remaining present for any questions.

**Table 1:** Cluster characteristics by region, IMD deciles are reported here and so range from 1 to 10, a lower IMD indicates higher deprivation

	**Number of schools** ***N (%)***	**IMD** ***mean (SD)***	**School size** ***mean (SD)***
London	22 (38.6)	3.4 (1.3)	297.8 (232.3)
Northwest	17 (29.8)	5.8 (3.4)	218.9 (93.5)
Southwest	9 (15.8)	7.0 (3.2)	211.3 (107.5)
Midlands	9 (15.8)	4.8 (3.4)	190.7 (77.5)
Overall	57 (100)	4.9 (3.0)	243.7 (164.9)

Prior to 3- and 6-month follow-up appointments, the participants were briefed by school staff (and by the RA at the start of the appointment) not to reveal the workshop allocation of the school in order to keep the RA blinded. To improve follow-up rates, vouchers were offered to participants for completion of assessments at post randomisation timepoints. If participants were absent on the day the assessments took place, the RA attempted to return to see them when they were available or sent an assessment pack out by post for completion and return.

### Sample size

The sample size required for this study was 900 participants recruited from 60 schools. This number accounts for an assumed loss to follow-up of 12.5% of students and 4% of schools. As this is a cluster randomised trial, the sample size estimation is inflated based off an intra-class correlation of 0.02 to account for similarities within cluster. A sample of this size would provide 90% power to detect a clinically meaningful effect size of 0.28 on the primary outcome the Mood and Feelings Questionnaire [24].

### Statistical analysis

The baseline characteristics of the schools and participants were summarised using appropriate summary statistics. Baseline measures of the primary and secondary outcomes were scored and summarised to characterise the sample. All data manipulation and summaries were performed using Stata version 18. The trial statistical analysis plan (SAP) can be found as supplementary material to the protocol [21]. The primary outcome was analysed using a multi-level multivariable regression fitting schools with a random intercept, and adjusting for pre-specified covariates and presenting the adjusted mean difference with 95% CI, *p*-value and intra-cluster correlation.

### Patient and public involvement

Adolescent PPI groups from the Anna Freud Centre in London were consulted to inform effective recruitment strategies. PPI members advised on the content and delivery of participant recruitment presentations to provide optimal clarity of trial information, ensure appropriateness for the target population, and maximise engagement of the presentations. Focus groups were also run with groups of adolescent and young adult males from two of the trial regions to understand improved strategies for engagement of boys [25]. The findings of these focus groups were used to inform recruitment approaches, in particular transparency and honesty around participation and presence of a male ‘role model’ during recruitment events.

## Results

**Fig. 2:**
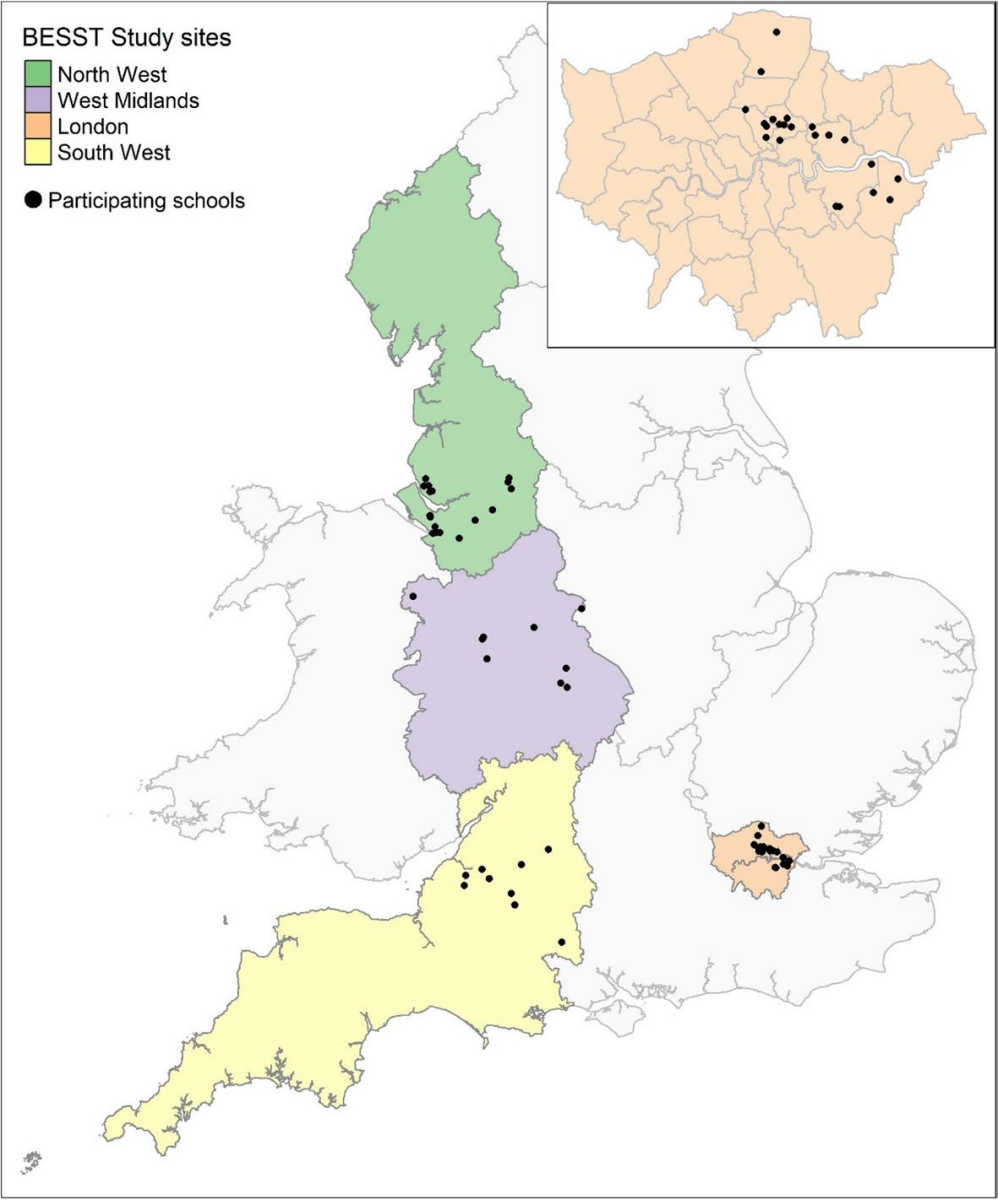
Map showing the locations of the 57 recruited schools across England

We recruited 900 participants from 57 schools into the study. School characteristics split by site are presented in Table 2. The majority of schools (39%) were recruited from London, and the average IMD of all 57 schools was 4.9 with average school size being 244 students. Figure 2 shows the location of all schools recruited into the study from the four sites. Approximate balance was seen across the two allocation arms for the minimisation factor within each region (Table 2).

**Table 2:** Participant baseline demographics

**Participant baseline demographics**	***N* = 900** ***N (%)***
Age, mean (SD)	17.2 (0.6)
Gender	
* Male*	228 (25.3)
* Female*	641 (71.2)
* Other*	21 (2.3)
* Prefer not to say*	10 (1.1)
Ethnicity	
* White*	468 (52.0)
* Mixed*	59 (6.6)
* Asian*	155 (17.2)
* Black*	141 (15.7)
* Chinese*	15 (1.7)
* Other*	41 (4.6)
* Missing*	21 (2.3)
Sixth form/college year	
Year 1	443 (49.2)
Year 2	454 (50.4)
Missing	3 (0.3)
Number of GCSEs passed, mean (SD)	8.7 (1.6)
Participant IMD, mean (SD)	4.6 (2.8)
English first language, yes	773 (85.9)
Previously sought help from GP for mental health, yes	179 (19.9)
Had counselling or talking therapy, yes	272 (30.2)
* Was this help through school, yes?*	183 (67.3)
* Was this help through CAMHS, yes?*	90 (33.1)
Was the study recommended by the teacher, yes	371 (41.2)

Baseline characteristics of participants show that the sample mean age was 17.2 (SD = 0.6) years old, is predominantly female (*n* = 641, 71%), white (*n* = 468, 52%) and spoke English as their first language (*n* = 772, 86%). In terms of ethnicity, 52% were white British, 45.6% were not white British, with the largest minority groups being Asian (17.2%) and Black (15.7%). The participant IMD mean was 4.6 (2.8) with a range of 1 to 10. In terms of gender, 228 male participants were recruited making up 25% of our sample.

Notably, most (80%) have never previously sought help from their GP for mental health or ever had previous counselling or talking therapy (70%) indicating that, although this population would like to receive help for stress, few had taken formal routes to acquire this. In terms of broader health and social care use, in the 3 months prior to study enrolment, 33% had seen a GP, 9% had seen a nurse, 6% had visited A&E, 6% had seen a school based mental health worker and 5% had seen a therapist outside of school. Participants were also asked to report any previous diagnoses for mental health conditions; 9% reported previously diagnosed anxiety disorder and 5% depression.

Summaries of the primary and secondary outcome measures are provided in Table 3. The established cut-off of 27 for symptoms of depression on the MFQ [26] was exhibited by 35% of the sample. The trial population also had an average *T*-score on the Revised Child Anxiety and Depression Scale (RCADS) in the normal range for anxiety [27], although 27% could be classed as sub-threshold or having symptoms of anxiety at baseline.

**Table 3:** Baseline participant level primary and secondary outcome summaries. A lower score indicates better clinical outcome for the MFQ and RCADS. A higher score indicates better clinical outcome for WEMWBS, Sleep condition indicator, CYRM-12 and EQ-5D-3L VAS (visual analogue scale). The CA-SUS and EQ-5D-3L will be analysed fully in the cost-effectiveness analysis, relevant summary information has been pulled from each measure for the purpose of characterising our sample. CA-SUS information refers to any use in the last 3 month, where numbers are very small <10 has been used instead to indicate low prevalence whilst still preserving participant anonymity

	**N**	**Mean (SD) or %**	**Interpretation **
**Primary outcome**			
Mood and Feelings Questionnaire (MFQ)	898	23.4 (12.4)	
≤ 27	584	65.0%	Indicates no presence of symptoms of depression
> 27	314	35.0%	Indicates the presence of symptoms of depression
**Secondary outcomes**			
Revised Child Anxiety and Depression Scale (RCADS)			
Anxiety raw score	895	41.4 (18.9)	
Anxiety T-score	864	55.5 (13.1)	
*< 65*	663	76.7%	Normal range
*between 65 and 69*	111	12.8%	Borderline clinical symptoms
*≥ 70*	125	13.9%	Clinical range
Warwick and Edinburgh Mental Wellbeing Scale (WEMWBS)	889	41.2 (8.8)	Range between 14 – 7016-18 population norm 49.2 (48.2 - 50.2)
*<49*	713	80.2%	
*≥ 49*	176	19.8%	
Sleep Condition Indicator	898	19.2 (6.7)	
*≤16*	314	35.0%	≤16 probable insomnia disorder
*> 16*	584	65.0%	
Child and Youth Resilience Measure (CYRM-12)	900	45.1 (7.7)	Range between 12 and 60
Child and Adolescent Service Use Schedule (CA-SUS)	900		
*Stayed overnight in hospital for mental health *	<10	<2%	
*Outpatient appointments for mental health*	26	2.9%	
*Visited A&E*	56	6.2%	
*GP appointment*	295	32.8%	
*Appointment with nurse in a GP practice or school nurse*	83	9.2%	
*CAMHS appointment*	25	2.8%	
*Therapist appointment outside of school*	43	4.8%	
*School based mental health worker appointment*	56	6.2%	
EQ-5D-3L VAS	899	67.6 (19.0)	Range between 0 and 100

The wellbeing of the sample measured using the Warwick and Edinburgh Mental Wellbeing Scale (WEMWBS) which showed that our sample had a lower average score for general wellbeing than the average score of 49 seen in 16–18-year-olds [28] with 80% scoring below the average. Overall, there was no indication of insomnia disorder on the sleep condition indicator [29]; however, 35% scored 16 or less which indicates that these participants may have probable insomnia disorder. In terms of the EQ-5D measure of health-related quality of life, the visual analogue scale score of 67.6 was also lower than the average utility values of 0.913 reported in UK 10–19-year-olds [30].

## Discussion

Recruiting schools can be very difficult but the strategies used in BESST, particularly collaborating with MHSTs have proven to be effective resulting in the target number of schools being recruited into the study. A key strength is that the study enrolment was to target both in terms of schools and participants, so the trial is adequately powered to detect a meaningful difference on the primary outcome measure if one exists.

Another strength of the study was the use of the self-referral system which is participant-led. As students were able to refer themselves without the barriers that can occur in access to services [20], under-served minority students were better recruited in this study. Among the marginalised groups (45.6%), the largest groups were Asian (17.2%) and black (15.7%). This study was also able to recruit those who had not previously sought help, with 80% never having sought help from their GP.

This study is well designed as selection bias was reduced as much as possible through the randomisation and baseline procedure. It is often difficult to avoid selection bias in cluster randomised trials as clusters must be randomised in blocks at the start of the study meaning that investigators are often aware of the allocation that the cluster has received whilst recruiting participants to that cluster. Schools were randomised in blocks and balanced on key covariates but allocations were only released to the trial team once all participants from that cluster had completed baseline assessment meaning that all participants were recruited into the study by researchers who were unaware of allocation so this could not influence recruitment in any way.

With the exception of gender, the sample appears to offer external validity as the baseline data resembles demographics and clinical characteristics that are reasonably representative of a typical school within our recruiting areas which have an average IMD of 5.5 with 33% of students being non-white over all 4 recruiting areas [31, 32]. A possible weakness of the study is the sample was predominantly female so offers a slight under representation of males, however less than 30% of males experience depression and anxiety problems [33], with 20% experiencing more behavioural problems.

Students put themselves forward for the study based on self-perceived need for help in dealing with stress. Given this, it was expected that students would report different degrees of depression and anxiety with some students scoring above threshold, some sub-threshold as well as those not reporting significant symptoms. The lower levels of general wellbeing indicate that there is a need for support, and that it was possible to recruit participants with indications of depression and anxiety without the need for strict eligibility criteria based on diagnostic symptoms which could lead to increased stigma within the school setting [14, 16].

## Conclusions

In conclusion, this study has been a well-designed study, which has succeeded in recruiting 900 participants from 57 schools over 2 years. Participants are representative of the school population and notably and unusually in research studies, just under half participants are from ethnic minorities (45.6%).

## Data Availability

The chief investigator will act as custodian of the data in accordance with legislation and the terms of the research sponsor (King's College London) and funder (National Institute for Health Research, UK).

## Abbreviations

MFQ: Mood and Feelings Questionnaire
WEMWBS: Warwick Edinburgh Mental Wellbeing Scale
CAMHS: Child and Adolescent Mental Health Services
CBT: Cognitive behavioural therapy
MHSTs: Mental Health Support Teams
RA: Research Assistant
IMD: Index of multiple deprivation
PPI: Patient and public involvement
RCADS: Revised Child Anxiety and Depression Scale
